# P03-027 - A brief Case report on specific undiagnosed condition, whose provisional diagnosis on the basis of provisional lab investigation proved as one of the component of autoinflammatory disorder

**DOI:** 10.1186/1546-0096-11-S1-A225

**Published:** 2013-11-08

**Authors:** S Sathyanandan, LK Md

**Affiliations:** 1 Pediatrics, SAT Hospital, Medical College Trivandrum, Kerala, India

## Introduction

The autoinflammatory syndromes are a newly recognized group of immune disorders that lack the high titers of self-reactive antibodies and T cells characteristic of classic autoimmune disease. , patients with these illnesses experience unprovoked inflammatory disease in the absence of underlying infection.(Curr Top Microbiol Immunol. 2006;305:127-60).

In the category of autoinflamation this disease is a a lesion particularly known as CRMO or chronic recurrent mutifocal osteomylitis a rare condition in which bones have an lesion, inflammation and pain but without a focus of infection .the result of its inheritance pattern is still being debated and the distinguishing feature from the autoimmune pattern is still not clear.

It is classified as an auto inflammatory disease due to its recurrent out breaks and lack of any known pathogen.this disease tends to range from 4 to 14 years of age and 10 years as median. As by current statistics it occur 1:1000000 and primarly in girls with a 5:1 ratio.

CRMO may similar to a painful bone lesion such as arthritis , RF , bacterial osteomylitis ,ewings sarcoma, lymphoma, eosinophilic granuloma or LAH. When all the prevoious illness are ruled out and a bone biopsy turns negative for any known cancer CRMO is usually diagnosed.

As such the common medication given for reducing the inflammation are such as NSAIDS and steroids, antibiotics are not prescribed as there is no evident focus of infection. Congenital dyserthropoitic anaemia and CRMO , uncommon childhood diseases an association with sweet syndrome may be interelated .( journal of paediatrics 115 (5, part 1):730-4. Dol10.1016/S0022-3476(89)80650.

A retrospective review of autoinflamatory diseases in Saudi children at rheumatology clinic showed that of the 34 children with a mean age 118 months , consanguinity was present in 40 %. And in which FMF 50%, CRMO 23.5%, EOS 8.8%, MWS 6 %.(Ann Saudi med . 2012 ;32(1);43-8(ISSN;0975-4466).

CRMO is sometimes associated with palmoplantar pustulosis and frequent relapses may be present with remission lasting for 4-16 weeks. The most distal case if the affected series . classical Xray diagnosis reveled skeletal changes consistent with appearance of acute or chronic heamoatogenic osteomylits.morphologically CRMO begins as an acute inflammatory process either predominance of PMN leucocytes, with occasionally form absesses and osteoclastic bone resorption. At a later stage it may present as lymphocytes and inflamatoty infiltrate and occasional granulomatous foci and signs of new bone formation.

## Case report

The pathological basis of the inflammation in the various inflammatory foci of bone lesions without the exact non infectious cause is much debated part in the pathogenesis of chronic recurrent multifocal osteomylitis and the recurrent attacks that are caused may be due to an inherent autoimmune part of the disease in the body.

To identify the pathophysiological cause of the recurrent inflammatory attacks on the bone with due consideration to the time period of each attack.

The specific treatment stratergy is presently soley based on steroid therapy and the judicious use of NSAIDs for each specific attack that is manifested therefore the need to design a protocol for the treatment of recurrent attacks so as to include immunomodulators in the list may be helpful in remission.

## Method

### Study design

A prospective cohort study with a chance of reverting back to double blinded Randomized control trial in due course may be considered.

### Study setting

SAT Hospital Trivandrum and a proposed teritiary care setting where the translational research could undertaken.

### Study period

18 May 2011 – Ongoing.

### Study subjects

A part from the patient , the specific disease has shown to have a familial inheritance pattern, so the genotypic variance along with a pedigree analysis is mandatory in establishing the pathophysiological cause ,so the near and distant family members might be also a part in as study subjectsA semistructured questionare was devised at the onset of study and progress of the disease is currently documented.

## Results

The brief outline of the natural history is being given as below. The patient of 9 years was brought to the orthopaedic Opd with first episode of recurrent pain and redness in the joint of the third interphalengial joint and in the knee joint of the upper tibia and the preliminary investigation done as of which is done in the teritiary care hospital . the routine Xray showed very well circumscribed callus around the lesion and the dull sclerosing opacities around the joint after which the it as subjected to open wide excision and sent for HP analysis. The result first showed inflammatory infiltrate and features suggestive of auto immune condition, it was also sent for AFB culture result of which came as negative.

On the provisional basis it was first diagnosis as garres osteomylitis but features of surrounding or well circumscribed marginal sclerosing lesions were present and as of which therapy was given with local antibiotics , the recurrent attacks in the due course made the the patient get refferd to pediatric OPD on complete review on an immunological basis . the ANA profile was done but the only sight fluctuations were seen in the markers. HLAB 27 was also performed which came as negative , rest of the immunological studies are currently proceded. The contrast enhanced MRI showe the features of suggestive of CRMO. As subjected to the low socioeconomic status of the patient higher investigations are pending, the current presisting symptom that the patient complain is the recurrent attacks of pain in at the lesion site. The two sites ie, the head of humerus and the and the tip of olecranon was not subjected to biopsy as it would hamper the epiphysial growth. The detailed presenting history, clinical examination, lab investigations and status of the subject cannot be revealed at present as the patient is only subjected to waiver of consent. As CRMO is diagnosis of Exclusion the next investigation most preferably would be A TPMT assay, laser cut biopsy of the specimen and detailed immunohistochemistry. the provisional diagnosis made were JIA, Chronic Non bacterial osteomylitis, Non Monogenic AIDs, systemic onsent of other autoimmune disease.

## Discussion

A detailed history of the Specfic Autoinflamatoty disorder.

## Disclosure of interest

None declared

